# Collagen I Increases Palmitate-Induced Lipotoxicity in HepG2 Cells via Integrin-Mediated Death

**DOI:** 10.3390/biom14091179

**Published:** 2024-09-20

**Authors:** Tumisang Edward Maseko, Eva Peterová, Moustafa Elkalaf, Darja Koutová, Jan Melek, Pavla Staňková, Veronika Špalková, Reem Matar, Halka Lotková, Zuzana Červinková, Otto Kučera

**Affiliations:** 1 Department of Physiology, Faculty of Medicine in Hradec Králové, Charles University, Šimkova 870, 500 03 Hradec Králové, Czech Republic; masekot@lfhk.cuni.cz (T.E.M.); elkalafm@lfhk.cuni.cz (M.E.); jan.melek@lfhk.cuni.cz (J.M.); stankovap@lfhk.cuni.cz (P.S.); spalkovve@lfhk.cuni.cz (V.Š.); matarr@lfhk.cuni.cz (R.M.); lotko@lfhk.cuni.cz (H.L.); wolff@lfhk.cuni.cz (Z.Č.); 2 Department of Medical Biochemistry, Faculty of Medicine in Hradec Králové, Charles University, Šimkova 870, 500 03 Hradec Králové, Czech Republic; peterove@lfhk.cuni.cz (E.P.); koutova.darja@lfhk.cuni.cz (D.K.)

**Keywords:** in vitro NAFLD models, collagen I, palmitate, lipotoxicity, α2β1 receptors, integrin-mediated death, HepG2 cells

## Abstract

Various strategies have been employed to improve the reliability of 2D, 3D, and co-culture in vitro models of nonalcoholic fatty liver disease, including using extracellular matrix proteins such as collagen I to promote cell adhesion. While studies have demonstrated the significant benefits of culturing cells on collagen I, its effects on the HepG2 cell line after exposure to palmitate (PA) have not been investigated. Therefore, this study aimed to assess the effects of PA-induced lipotoxicity in HepG2 cultured in the absence or presence of collagen I. HepG2 cultured in the absence or presence of collagen I was exposed to PA, followed by analyses that assessed cell proliferation, viability, adhesion, cell death, mitochondrial respiration, reactive oxygen species production, gene and protein expression, and triacylglycerol accumulation. Culturing HepG2 on collagen I was associated with increased cell proliferation, adhesion, and expression of integrin receptors, and improved cellular spreading compared to culturing them in the absence of collagen I. However, PA-induced lipotoxicity was greater in collagen I-cultured HepG2 than in those cultured in the absence of collagen I and was associated with increased α2β1 receptors. In summary, the present study demonstrated for the first time that collagen I-cultured HepG2 exhibited exacerbated cell death following exposure to PA through integrin-mediated death. The findings from this study may serve as a caution to those using 2D models or 3D scaffold-based models of HepG2 in the presence of collagen I.

## 1. Introduction

The global rise of nonalcoholic fatty liver disease (NAFLD) over the past decades has prompted researchers to invest more effort in developing reliable models, including in vitro models, that could unravel the unknown intricate mechanisms of this disease and lead to the development of effective therapies [1]. NAFLD includes a spectrum of histological findings ranging from simple steatosis to nonalcoholic steatohepatitis (NASH), which may lead to fibrosis and hepatocellular carcinoma [2]. Various strategies have been employed to improve the reliability of 2D, 3D, and co-culture in vitro models of NAFLD, including using extracellular matrix proteins such as collagen I to mimic cell-to-extracellular matrix interactions [3,4,5]. Although it is generally known that the extracellular matrix promotes cell adhesion, viability, differentiation, and proliferation in most cells [6,7], information regarding the effects of using collagen I to develop 2D or 3D in vitro NAFLD models is insufficient. Understanding the impact of collagen I in 2D in vitro models is essential before it is widely adopted for scaffold-based 3D in vitro NAFLD models.

As alternatives for using primary human hepatocytes, HepG2 and HepaRG cells are the most utilized cell lines for in vitro models of NAFLD [1,8,9,10]. Although HepG2 has limitations such as low mitochondrial respiration and low expression of some nuclear receptors necessary for lipid metabolism [11], it exhibits findings similar to those in patients with late-stage NASH following exposure to palmitate (PA), e.g., increased hepatocyte apoptosis and decreased respiration [12,13,14]. Similarly, our recently published study demonstrated that exposure of HepG2 to free fatty acids resulted in altered mitochondrial morphology coupled with decreased respiration and increased ROS production [15]. Following the observation that exposure of HepG2 to PA reduced cell adhesion to cell culture vessels, resulting in loss of cells, it was hypothesized that culturing the cells on collagen I could improve cell adhesion and reduce PA-induced cell death. A study by Chethikkattuveli et al. [16] previously demonstrated that HepG2 cell adhesion was significantly enhanced by culturing it on collagen I. Collagen I is the most abundant extracellular protein in the human body and one of the most highly utilized extracellular matrices for cell culture [17]. Collagen I is a 300 kDa molecule composed of two alpha-1 chains and one alpha-2 chain that combine to form a triple helix scaffold [17]. Cells use various cell adhesion receptors, such as integrins, to bind to distinct types of collagens [6]. Integrin α2β1 is the primary receptor that binds collagen I and is present in most epithelial cells [18]. Previous studies have demonstrated that HepG2 cells cultured on collagen I exerted high proliferation correlated with increased expression of α2β1 receptors [19,20]. Although ligation of α2β1 receptors by collagen I does support the abovementioned advantages, unligated or antagonized α2β1 receptors have been found to activate apoptosis via integrin-mediated death [21,22]. While studies have demonstrated the significant benefits of culturing cells on collagen I as part of 2D or 3D in vitro platforms, its effects on HepG2 after exposure to PA have not been investigated. Patients diagnosed with NASH usually have a high serum concentration of PA [23]. Thus, PA is commonly used to develop NAFLD/NASH in vitro models [24,25].

This study aimed to assess the effects of PA-induced lipotoxicity in HepG2 cultured in the absence or presence of collagen I. Understanding the interactions of collagen I with this commonly utilized cell line may enable informed choices of whether to use collagen I to develop 2D or scaffold-based 3D in vitro NAFLD models.

## 2. Materials and Methods

### 2.1. Cell Culture

HepG2 cells (ECACC85011430, liver cancer cells) were purchased from ECACC and cultured in Minimum Essential Medium (Merck & Co., Inc., Rahway, NJ, USA) supplemented with 1% non-essential amino acids, 10% fetal bovine serum, a 1% mixture of penicillin (10,000 UI/mL) and streptomycin (10 mg/mL), and 1% sodium pyruvate. The cells were incubated at 37 °C in a 5% CO_2_, 95% air-humidified atmosphere, and passaged once a week at 75% confluency. HepaRG cells (HRP101, liver cancer cells) were purchased from Biopredic International and cultured at the recommended 26,600 cells/cm^2^ density. HepaRG was cultured in proliferation medium (William’s E Medium (Lonza Group, Ltd., Basel, Switzerland) supplemented with 5 μg/mL insulin, 50 μM hydrocortisone, 1% L-glutamine, 1% mixture of penicillin (10,000 UI/mL), streptomycin (10 mg/mL), and 10% fetal bovine serum) for 14 days. After that, they were cultured in a differentiation medium (proliferation medium supplemented with 1.5% dimethyl sulfoxide) for another 14 days. They were incubated at 37 °C in a 5% CO_2_, 95% air-humidified atmosphere, and the medium was changed three times a week.

On the day of seeding, cell culture vessels were coated using collagen I (rat tail, Merck & Co., Inc.). Collagen I stock solution (300 μg/mL) was prepared in 0.02 M acetic acid and allowed to dissolve for 24 h. Cell culture vessels were coated with collagen I for 30 min at 37 °C. After coating the cell culture vessels, collagen I was aspirated and neutralized with phosphate-buffered saline (PBS) and allowed to dry for 30 min at 37 °C before seeding the cells (HepG2 or HepaRG). The final concentration of collagen I used for coating was 5 μg/cm^2^. The cells were seeded for 24 h in the absence or presence of collagen I before applying treatments.

### 2.2. Preparation of Palmitate

Sodium palmitate (PA) was purchased from Merck & Co., Inc. As previously described [26], a 40 mM stock solution of PA was first prepared in 0.1 M NaOH (Merck & Co., Inc.), followed by conjugation to bovine serum albumin (BSA) (Merck & Co., Inc.). PA was dissolved at 70 °C for 30 min and stored at −80 °C for up to 3 months. To prepare the PA-BSA conjugation, 40 mM stock solution PA was dissolved and mixed with 20% BSA for 1 h to yield an 8 mM stock solution (pH 7.4), which was further dissolved in the culture medium (without fetal bovine serum) to yield the 1 mM final concentration needed to treat cells. The 8 mM stock solution was sterile-filtered prior to use. The molar ratio between PA and BSA was 5.3, and 2.5% BSA was used as the control [26]. Unless otherwise indicated, the cells were treated with 2.5% BSA and 1 mM PA for 8 h (total culture of 32 h) and 24 h (total culture of 48 h) (Figure S1A) in all the methods described below. Where necessary, detached cells were collected from the culture medium at 940× *g*, 4 °C, 5 min.

### 2.3. Cell Viability/Proliferation Assay

Cell viability and proliferation were determined using tetrazolium salt WST-1 (4-[3-(4-iodophenyl)-2-(4-nitrophenyl)-2H-5-tetrazolio]-1,3-benzene disulfonate) purchased from Roche, Ltd. (Basel, Switzerland). The assay is based on the cleavage of the slightly red tetrazolium salt WST-1 to form a yellow formazan dye by metabolically active cells. The WST-1 test was performed according to the manufacturer’s recommendations. Briefly, cells cultured in 96-well plates were treated with 10% WST-1 (diluted in cell culture medium), and absorbance (440 nm) was measured at 0 and 60 min using Tecan Infinite M200 (Tecan Group, Ltd., Männedorf, Switzerland).

### 2.4. Cell Adhesion, Proliferation, and Cytotoxicity

The xCELLigence real-time cell analysis (RTCA) system (Agilent Technologies, Inc., Santa Clara, CA, USA) measured cell impedance and automatically calculated cell index values, which provided information about cell adhesion, proliferation, and cytotoxicity. Half of the E-plate (96-well plate) was coated with collagen I, and after carrying out cell density titration measurements, the optimal seeding number of 10,000 cells per well was determined. After seeding, the E-plates were loaded on the RTCA station and placed in a CO_2_ incubator at 37 °C for 24 h. Following 24 h of attachment, the culture medium was removed. The cells were treated with a culture medium containing 2.5% BSA and 1 mM PA, and cell impedance was continuously measured for the next 4 days. The cells treated with 5% DMSO were used as positive control. Evaluations were performed using xCELLigence 1.2.1 software (Agilent Technologies, Inc.).

### 2.5. Mitochondrial Respiration

The extracellular flux analyzer Seahorse XFe-96 (Agilent Technologies, Inc.) measured oxygen consumption rate (OCR) in real time and provided information on mitochondrial respiration. Before the measurement, the culture media containing treatments (2.5% BSA and 1 mM PA) were replaced with assay medium (bicarbonate-free XF DMEM pH 7.4 (Agilent Technologies, Inc.) supplemented with 4 mM L-glutamine, 1 mM pyruvate, and 1 g/l D-glucose) and the cells were incubated in a CO_2_-free incubator for 1 h at 37 °C. The seeding density was 20,000 cells per well (96-well plates). Following the measurement of basal respiration, a mitochondrial stress test was performed by sequential additions of 1 μM oligomycin, 1.2 μM carbonyl cyanide-4-(trifluoromethoxy)phenylhydrazone (FCCP), and 1 μM rotenone and antimycin A. Differences in OCR values in response to respiratory modulators were used to calculate various mitochondrial parameters (basal and maximal respiration, ATP-linked respiration, spare respiratory capacity, and proton leak respiration); however, only maximal respiration was illustrated. Unless stated otherwise, the materials used here were purchased from Merck & Co., Inc.

### 2.6. Caspase Assays

Apoptosis was determined by monitoring the activities of caspases 3/7, caspase 8, and caspase 9 (Caspase-Glo Assays, Promega Corporation, Madison, WI, USA). The assays were used per the manufacturer’s recommendations at 32 and 48 h of culture. The caspase-Glo Reagent results in cell lysis, followed by caspase cleavage of the substrates and the generation of luminescent signals. After 1 h of incubation at room temperature (in the dark), luminescence from all the substrates was measured using Tecan Infinite M200 (Tecan Group, Ltd.).

In other experiments, caspase 3 activity was measured using a fluorescent probe (Ac-DEVD-AMC, Life Sciences, Inc., St. Petersburg, FL, USA). The cells were lysed in lysis buffer (50 mM HEPES, 5 mM CHAPS, and 5 mM DTT) and stored at −80 °C. The cell culture medium was also collected and stored at −80 °C. Samples were quantified in assay buffer containing 20 mM HEPES, 0.1% CHAPS, 5 mM DTT, 2 mM EDTA, and Ac-DEVD-AMC. Activated caspase enzymes cleave the probe to release fluorescent 7-amino-4-methylcoumarin (λex = 360 nm, λem = 465 nm), which was detected using Tecan Infinite M200 (Tecan Group, Ltd.). Unless stated otherwise, the materials used here were purchased from Merck & Co., Inc. 

### 2.7. Lactate Dehydrogenase (LDH) Assay

To evaluate plasma membrane integrity, the LDH assay kit (Diagnostic Systems GmbH) was used to quantify LDH activity (Tecan Infinite M200, Tecan Group, Ltd.) in cell culture medium and cell lysates according to the manufacturer’s instructions. LDH leakage (%) was then calculated from the measured LDH activities.

### 2.8. Reactive Oxygen Species (ROS) Production

CM-H_2_DCFDA (Invitrogen, Carlsbad, CA, USA; Thermo Fisher Scientific, Inc., Waltham, MA, USA) was used to evaluate the increased production of ROS. Its acetate groups are cleaved by intracellular esterases followed by subsequent oxidation, yielding a fluorescent adduct (λex = 485 nm, and λem = 535 nm) that becomes trapped inside the cells. In brief, the HepG2 cells were incubated with 40 μM of the indicator at room temperature for 30 min. The cells were washed with phosphate-buffered saline, and fluorescence was quantified using Tecan Infinite M200 (Tecan Group, Ltd.). Data were normalized to the protein concentration (BCA assay, Thermo Fisher Scientific, Inc.).

### 2.9. Enzyme-Linked Immunosorbent Assay (ELISA)

To quantify the integrin receptor subunit (ITGA2), the cells were lysed in RIPA lysis buffer (Merck & Co., Inc.). To quantify phosphorylated focal adhesion kinase (P-FAK), the cells were lysed in the recommended buffer (Invitrogen; Thermo Fisher Scientific, Inc.), consisting of 10 mM Tris (pH 7.4), 100 mM NaCl, 1 mM EDTA, 1 mM EGTA, 1 mM NaF, 20 mM Na_4_P_2_O_7_, 2 mM Na_3_VO_4_, 1% Triton X-100, 10% glycerol, 0.1% SDS, 0.5% deoxycholate, 1 mM PMSF, and protease inhibitor cocktail. All the ELISA kits were purchased from Invitrogen Thermo Fisher Scientific, Inc., and were used according to the manufacturer’s recommendations. TECAN Infinite M200 (Tecan Group, Ltd.) was used to measure absorbance at 450 nm. The data were normalized to protein concentration using the Bradford assay (Thermo Fisher Scientific, Inc.). Unless stated otherwise, the materials used here were purchased from Merck & Co., Inc.

### 2.10. RNA Isolation and Reverse Transcription–Quantitative Polymerase Chain Reaction

Total cellular RNA was extracted using TRIzol reagent (Invitrogen; Thermo Fisher Scientific, Inc.) in accordance with the manufacturer’s methodology. The isolated RNA was reverse-transcribed into complementary DNA (cDNA) using a cDNA Reverse Transcription Kit (Applied Biosystems, Waltham, MA, USA; Thermo Fisher Scientific, Inc.). Quantification of individual gene expressions was performed using TaqMan Gene Expression Assays. The list of used gene expression assays includes the following: Apoptosis regular BAX (BAX) Hs00180269_m1, BCL2 apoptosis regular (BCL2) Hs04986394_s1, BH3-interacting domain death agonist (BID) Hs00609632_m1, cyclin-dependent kinase inhibitor 1B (CDKN1B) Hs00153277_m1, Fas-associated death domain (FADD) Hs00538709_m1, integrin subunit alpha 2 (ITGA2) Hs00158127_m1, integrin subunit beta 1 (ITGB1) Hs01127536_m1, PPARG coactivator 1 alpha (PPARGC1A) Hs00173304_m1, and tumor protein p53 (TP53) Hs_00153349_m1. Gene expression analysis was performed using the Quant studio 6 Real-Time PCR system (Applied Biosystems; Thermo Fisher Scientific). The gene expression analysis results were normalized to RNA polymerase II subunit A (POLR2A) (Hs00172187_m1) RNA expression. Gene expression levels were calculated using a comparative Ct (ΔΔCt) method.

### 2.11. Statistical Analysis

All experiments consisted of a minimum of three independent replicates. Statistical analysis was performed using GraphPad Prism 9.2.0 (GraphPad Software Inc., San Diego, CA, USA). Data are expressed as the mean ± SD. Following normality tests, two-way ANOVA followed by Tukey’s post hoc multiple comparisons test was used to assess significance among experimental groups. A *p*-value < 0.05 was considered to indicate a statistically significant difference.

## 3. Results

### 3.1. Culturing HepG2 Cells on Collagen I Is Associated with Increased Cell Proliferation, Adhesion, and Expression of α2β1receptors, and Improved Cellular Spreading

**Figure 1:**
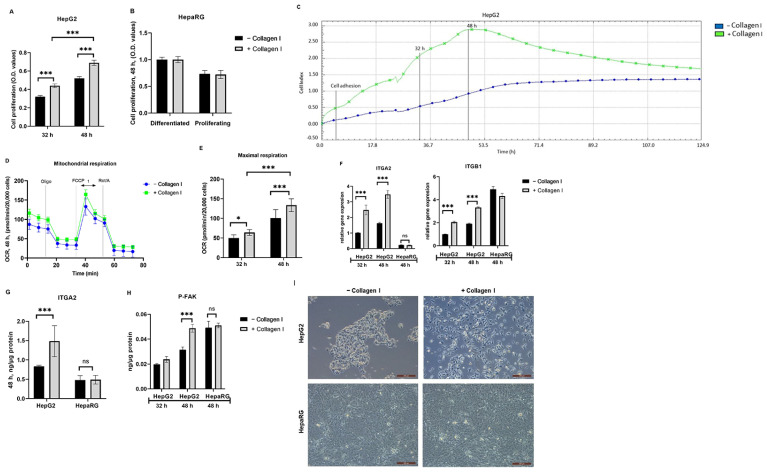
Effects of culturing HepG2 and HepaRG in the absence or presence of collagen I for 32 and 48 h. A total of 32 h of culture is composed of 24 h attachment and 8 h of 2.5% BSA. A total of 48 h of culture is composed of 24 h attachment and 24 h of 2.5% BSA. Cell proliferation in (**A**) HepG2 and (**B**) differentiated and proliferating HepaRG. (**C**) Cell adhesion and proliferation in HepG2 were measured using the xCELLigence real-time cell analysis system for a period of five days. The plots shown are representative of at least three replicate experiments. (**D**) Mitochondrial respiration in HepG2 after 48 h of seeding was measured using a Seahorse XFe96 real-time extracellular flux analyzer. (**E**) Maximal respiration [1] in HepG2 was calculated from the oxygen consumption rate measured using a Seahorse XFe96 real-time extracellular flux analyzer. (**F**) Relative gene expression of integrin receptor subunits (ITGA2 and ITGB1). Protein expression of (**G**) the integrin receptor subunit (ITGA2, 48 h) and (**H**) phosphorylated focal adhesion kinase (P-FAK) were quantified using enzyme-linked immunosorbent assay. (**I**) Images showing cell morphologies of HepG2 and HepaRG in the absence or presence of collagen I were captured using a light microscope (objective magnification, x10). Data are expressed as mean ± SD. Statistical analyses were carried out using two-way ANOVA followed by Tukey’s post-hoc test. * *p* < 0.05, *** *p* < 0.001. ns, not significant. (**A**,**B**) *n* = 8, (**D**,**E**) *n* = 16, and (**F**–**H**) *n* = 3. O.D: optical density, Oligo: oligomycin, FCCP: carbonyl cyanide-p-trifluoromethoxyphenylhydrazone, Rot: rotenone, A: antimycin A. BSA: bovine serum albumin. −Collagen I: absence and +Collagen I: presence of collagen I.

Cell proliferation assay was used to confirm whether culturing HepG2 and HepaRG cells on collagen I increases proliferation as previously described [19]. It was found that collagen I-cultured HepG2 cells significantly increased proliferation compared to those cultured in the absence of collagen I in a time-dependent response (*p* < 0.001) (Figure 1A). Interestingly, there was no significant difference in the proliferation of HepaRG cells irrespective of the absence or presence of collagen I (Figure 1B); therefore, they served as a vital control.

In addition, the xCELLigence system measuring cell impedance in real time was used to assess cell adhesion and proliferation of HepG2 cells cultured for five days. The cell-index values from xCELLigence RTCA further demonstrated that cell adhesion and proliferation in collagen I-cultured HepG2 cells were higher (3-fold higher after 48 h) than those cultured in the absence of collagen I (Figure 1C). Cellular respiration may serve as an essential surrogate for cell viability and proliferation. Therefore, the Seahorse XFe96 real-time extracellular flux analyzer was used to measure the OCR in collagen I-cultured HepG2 cells and those cultured in the absence of collagen I. As shown in Figure 1D,E, OCR values in collagen I-cultured HepG2 cells were significantly higher than those cultured in the absence of collagen I, and the differences were time-dependent (32 and 48 h, *p* < 0.001).

The signaling pathway described in the literature involving collagen I, cell proliferation, integrin receptors, and downstream activation of P-FAK [6] was evaluated. FAK is a cytoplasmic protein tyrosine kinase involved in integrin-mediated signal transduction regulating cell adhesion and proliferation [6]. As shown in Figure 1F,G, compared to HepG2 cultured in the absence of collagen I, HepG2 cultured on collagen I exhibited increased gene and protein expressions of integrin receptor subunits ITGA2 (*p* < 0.001) and gene expression of ITGB1 (*p* < 0.001) (protein expression was not evaluated). On the contrary, HepaRG exhibited no changes in gene and protein levels of ITGA2 or gene expression of ITGB1, irrespective of the absence or presence of collagen I. Accordingly, in comparison to HepG2 cultured in the absence of collagen I, HepG2 cultured in the presence of collagen I exhibited significantly high levels of P-FAK (48 h, *p* < 0.001) (Figure 1H). In accordance with data from HepaRG, the levels of P-FAK were the same irrespective of the absence or presence of collagen I. Images from a light microscope were captured to further confirm cell number and morphological changes due to culturing cells on collagen I. HepG2 cultured on collagen I were numerous, showed improved cell spreading, and formed elongated projections, while HepG2 cultured in the absence of collagen I tended to grow in clusters (Figure 1I). By contrast, as shown in Figure 1I, HepaRG cultured in the presence or absence of collagen I did not show differences in number or morphology.

### 3.2. PA-Induced Lipotoxicity Is Greater in Collagen I-Cultured HepG2 Cells than in Those Cultured in the Absence of Collagen I and Is Associated with Increased α2β1 Receptors

**Figure 2:**
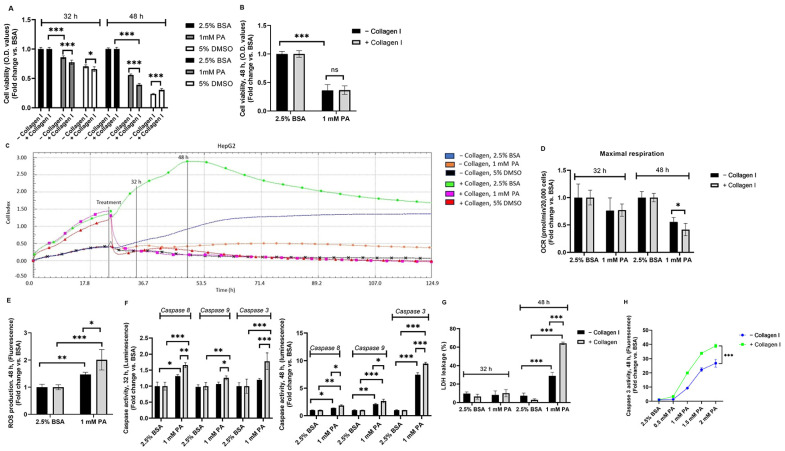
Lipotoxic effects of palmitic acid (PA) in HepG2 and HepaRG cultured in the absence or presence of collagen I for 32 and 48 h. A total of 32 h of culture is composed of 24 h attachment and 8 h of 2.5% BSA or PA. A total of 48 h of culture is composed of 24 h attachment and 24 h of 2.5% BSA or PA. Cell viability in (**A**) HepG2 (5% DMSO was used as a positive control) and (**B**) HepaRG (48 h). (**C**) Cell adhesion, proliferation, and detachment in HepG2 were measured using the xCELLigence real-time cell analysis system for a period of five days. The plots shown are representative of at least three replicate experiments. (**D**) Maximal respiration in HepG2 was calculated from the oxygen consumption rate measured using a Seahorse XFe96 real-time extracellular flux analyzer. (**E**) Reactive oxygen species (ROS) production following 48 h of culture. (**F**) Caspase activities (Luminescence) in HepG2. (**G**) Percent (%) of lactate dehydrogenase (LDH) leakage in HepG2. (**H**) Caspase 3 activity (48 h fluorescence) in the cell culture medium of HepG2 following exposure to different concentrations of PA (0.5, 1, 1.5, and 2 mM). Data are expressed as mean ± SD. Statistical analyses were carried out using two-way ANOVA followed by Tukey’s post-hoc test. * *p* < 0.05, ** *p* < 0.01, *** *p* < 0.001. ns, not significant. (**A**–**C**) *n* = 8, (**D**) *n* = 16, and (**E**–**H**) *n* = 3. O.D: optical density, BSA: bovine serum albumin. −Collagen I: absence and +Collagen I: presence of collagen I.

Having established the effects of the presence of collagen I in HepG2 and HepaRG, this study assessed cell viability following exposure to 1 mM PA for 8 and 24 h. Compared to controls (2.5% BSA, absence or presence of collagen I) in both cell lines (HepG2 and HepaRG), PA-treated cells exhibited significantly decreased cell viability irrespective of the absence or presence of collagen I (*p* < 0.001) (Figure 2A,B). Notably, contradictory to our expectations, it was found that collagen I-cultured HepG2 cells exerted significantly decreased cell viability compared to HepG2 cultured in the absence of collagen I after exposure to 1 mM PA in a time-dependent response (*p* < 0.001) (Figure 2A). On the other hand, there was no significant difference in cell viability between collagen I-cultured HepaRG cells and those cultured in the absence of collagen I after exposure to 1 mM PA (Figure 2B). Since decreased cell viability may correlate with reduced cell adhesion [27,28], xCELLigence RTCA was used to continuously monitor cell proliferation and adhesion of HepG2 cultured in the absence or presence of collagen I for 5 days. The findings from xCELLigence RTCA confirmed a time-dependent response that PA-treated HepG2 on collagen I had reduced cell adhesion than PA-treated HepG2 in the absence of collagen I, as shown in Figure 2C. Decreased cell viability due to PA exposure also correlates with reduced oxygen consumption [26]; thus, mitochondrial respiration was assessed in PA-treated HepG2 cells cultured in the absence or presence of collagen I. There were no significant differences in maximal respiration after 32 h of culture between PA-treated HepG2 cells cultured in the absence or presence of collagen I. However, at 48 h, PA-treated HepG2 cells cultured in the presence of collagen I exerted significantly lower maximal respiration than those cultured in the absence of collagen I (*p* < 0.05) (Figure 2D). As mitochondria play a significant role in ROS production [29], mitochondrial damage after 48 h of culture was further confirmed by data showing increased ROS production in PA-treated HepG2 irrespective of the absence or presence of collagen I (Figure 2E). Accordingly, ROS production was significantly higher in PA-treated HepG2 on collagen I than in that cultured in the absence of collagen I (*p* < 0.05) (Figure 2E).

Following the unexpected findings from PA-treated HepG2 cells cultured on collagen I, it was hypothesized that integrin-mediated death could be responsible for the effect. Therefore, caspase activities (especially caspase 8 activity, as it plays a vital role in integrin-mediated death [30]), LDH leakage, expression of pro- and anti-apoptotic genes (BID, BCL2, BAX, FADD, TP53), a cell cycle-regulating gene (CDKN1B), protein (ITGA2) and gene expressions of α2β1 receptor, protein level of P-FAK, and mitochondrial biogenesis regulating gene (PPARGC1A) were assessed. As shown in Figure 2F, compared to the controls, PA-treated HepG2 exhibited significantly increased caspase 8, 9, and 3 activities irrespective of the absence or presence of collagen I (except for caspase 9 activity after 32 h of culture in PA-treated HepG2 in the absence of collagen I). Similar to findings concerning cell viability, it was found that collagen I-cultured HepG2 exerted significantly higher caspase 3 activity compared to HepG2 cultured in the absence of collagen I after exposure to 1 mM PA in a time-dependent response (Figure 2F). Affirmingly, as shown in Figure 2F, after 32 h of culture, caspase 8 activity was significantly higher in PA-treated HepG2 on collagen I than in PA-treated HepG2 in the absence of collagen I. LDH leakage was measured at 32 and 48 h of culture to determine whether cell necrosis (LDH leakage through the cell membrane) was part of the cell death response at these time points. It was found that short-term (8 h) exposure to PA did not result in significant LDH leakage in both PA-treated HepG2 groups in the absence or presence of collagen I (Figure 2G). However, long-term (24 h) exposure to PA resulted in significantly increased LDH leakage in both PA-treated HepG2 groups (absence or presence of collagen I). Accordingly, LDH leakage was also significantly higher in PA-treated HepG2 on collagen I than in that cultured in the absence of collagen I (*p* < 0.001) (Figure 2G). To evaluate whether the difference in PA-induced cell death between HepG2 cultured in the absence or presence of collagen I was also dose-dependent, caspase 3 activity was measured in the cell culture medium following 0.5, 1, 1.5, and 2 mM PA exposure (Figure 2H). It was found that caspase 3 activity increased in a dose-dependent response in PA-treated HepG2 cells irrespective of the absence or presence of collagen I. Accordingly, caspase 3 activity in PA-treated HepG2 cells on collagen I was significantly higher than those cultured in the absence of collagen I (*p* < 0.001) (Figure 2H).

**Figure 3:**
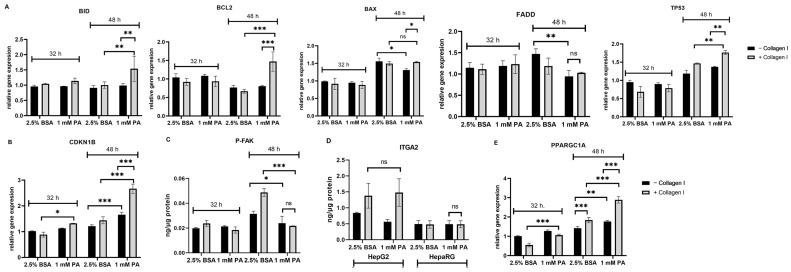
Effects of palmitic acid (PA) in the gene and protein levels of HepG2 and HepaRG cultured in the absence or presence of collagen I for 32 and 48 h. A total of 32 h of culture is composed of 24 h attachment and 8 h of 2.5% BSA or PA. A total of 48 h of culture is composed of 24 h attachment and 24 h of 2.5% BSA or PA. Relative gene expression of pro-apoptotic and anti-apoptotic genes (**A**) BID, BCL2, BAX, FADD, TP53 and (**B**) CKN1B in HepG2. Protein expression of (**C**) phosphorylated focal adhesion kinase (P-FAK, 48 h) in HepG2 and (**D**) integrin receptor subunit (ITGA2) in HepG2 and HepaRG was quantified using enzyme-linked immunosorbent assay. (**E**) Relative gene expression of mitochondrial biogenesis-regulating gene (PPARGC1A) in HepG2. Data are expressed as mean ± SD. Statistical analyses were carried out using two-way ANOVA followed by Tukey’s post-hoc test. * *p* < 0.05, ** *p* < 0.01, *** *p* < 0.001. ns, not significant. (**A**–**E**) *n* = 3. BSA: bovine serum albumin. −Collagen I: absence and +Collagen I: presence of collagen I.

The findings from gene expression studies generally show that pro- and anti-apoptotic genes were not significantly affected after 32 h of culture, as shown in Figure 3A. On the other hand, there were some statistically significant differences concerning pro- and anti-apoptotic genes between PA-treated HepG2 in the presence or absence of collagen I after 48 h of culture (except for FADD gene expression). Notably, gene expression of BID (substrate for activated caspase 8 [31]) was significantly higher in PA-treated HepG2 on collagen I than in that cultured in the absence of collagen I (Figure 3A). In addition, as shown in Figure 3B, gene expression of the cell cycle inhibitor (CDKN1B) was also significantly greater in PA-treated HepG2 cells on collagen I than in those cultured in the absence of collagen I, even after 32 h of culture. CDKN1B acts downstream of FAK [32]. Subsequently, this study assessed the protein levels of P-FAK following exposure to PA in HepG2 (absence or presence of collagen I) (Figure 3C). It was revealed that PA-treated HepG2 cells on collagen I significantly reduced levels of P-FAK compared to the control (48 h) (*p* < 0.001). In comparison, PA-treated HepG2 cultured in the absence of collagen I had slightly decreased levels of P-FAK compared to the control (48 h) (*p* < 0.05). As α2β1 receptors were hypothesized to be involved in the effects seen in this study, the levels of the integrin receptor subunits were evaluated following PA exposure. Our findings show that in comparison to the control, the protein levels of ITGA2 (there was a decrease in gene expression, as shown in Figure S1B) and gene expression of ITGB1 did not change in PA-treated HepG2 cultured on collagen I (Figure 3D and Figure S1B). Similarly, the protein levels of ITGA2 were also not altered by PA exposure in HepaRG (Figure 3D). Moreover, since mitochondrial damage can be associated with compensatory mitochondrial proliferation [15,33], gene expression of PPARGC1A was quantified. Gene expression of PPARGC1A was significantly higher in PA-treated HepG2 cells on collagen I after 48 h than in those cultured in the absence of collagen I (Figure 3E).

To further confirm the role played by caspases in exacerbating PA-induced cell death in HepG2 on collagen I, cell viability was measured following coincubation of 1 mM PA with caspase 8, 9, and 3 inhibitors at 32 and 48 h of culture (Figure S1C). Disappointingly, as reported elsewhere [34,35], it was found that caspase inhibitors could not significantly decrease reduced cell viability caused by PA exposure, irrespective of the absence or presence of collagen I (Figure S1C).

Finally, triacylglycerol (TAG) accumulation was quantified to assess its role in the findings of this study. As shown in Figure S1D, the TAG content was significantly increased after PA exposure in both conditions (absence or presence of collagen I). Nonetheless, there were no differences in the TAG content of PA-treated HepG2 cultured in the absence or presence of collagen I.

## 4. Discussion

Collagen I is one of the most used cell adhesion materials in cell culture, and its utility has significantly increased owing to the need to improve 2D models and increased recommendations for transformation to 3D models [5,17]. This study assessed PA-induced cell death in HepG2 cells cultured in the absence or presence of collagen I. For the first time, this study revealed that culturing HepG2 cells in the presence of collagen I was associated with increased PA-induced cell death compared to culturing in the absence of collagen I.

Numerous ways have been adopted to improve 2D models to be more suitable for NAFLD in in vitro studies—for example, using coculture 2D models, selecting suitable primary cells and cell lines, and enhancing cell adhesion and extracellular communication of utilized cells [1,4]. Collagen I-sandwiched primary rat hepatocytes maintain the secretion of important hepatic markers, such as fibrinogen, albumin, and bile acids, for up to 6 weeks [36]. Three-dimensional cell cultures of HepG2 cells using collagen I have also shown increased albumin and urea secretion and higher expression/activities of some xenobiotic-metabolizing enzymes [37,38]. Consistent with the previous studies [19,20], the current study also demonstrated that culturing HepG2 cells in the presence of collagen I resulted in increased cell adhesion, which was correlated with increased cell proliferation and mitochondrial respiration, higher levels of integrin receptors and P-FAK, and improved cell spreading. The findings from HepG2 cells cultured on collagen I were further validated by the data from HepaRG cells, showing that a lack of enhanced cell proliferation on collagen I was correlated with unchanged levels of integrin receptors and P-FAK, and no change in cellular morphology. Having confirmed the benefits of culturing HepG2 in the presence of collagen I, it was hypothesized that seeding HepG2 on collagen I could improve cell adhesion and reduce PA-induced cell death. To test our hypotheses, this study measured and compared various markers of cell death (cell viability, cell adhesion, caspase activities, LDH leakage, pro- and anti-apoptotic genes, and gene expression of CNKN1B and TP53), mitochondrial respiration and biogenesis, P-FAK, gene and protein levels of integrin receptor subunits, ROS production, and TAG accumulation in PA-treated HepG2 cultured in the absence or presence of collagen I. Some of the parameters mentioned above were also assessed in PA-treated HepaRG cultured in the absence or presence of collagen I.

In general, the findings concerning lipotoxicity due to PA exposure in HepG2 and HepaRG, irrespective of the absence or presence of collagen I, were consistent with previous in vitro studies or clinical studies of late stages of NASH, demonstrating increased cell death, decreased mitochondrial respiration, and high ROS production [12,13,14,39]. However, contradictory to our expectations, these parameters were all exacerbated in HepG2 cultured in the presence of collagen I. These unexpected findings demanded a possible explanation; therefore, another hypothesis was formulated. After considering all the data from HepG2 and HepaRG, we hypothesized that increased cell death in HepG2 cultured in the presence of collagen I was due to integrin-mediated death. Previous studies have demonstrated that unligated or antagonized integrins recruit caspase 8 to the cell membrane and activate apoptosis through integrin-mediated death [21,30,40]. Noting the correlation between increased activity of caspase 8 in PA-treated HepG2 cells cultured on collagen and high levels of the α2β1 receptors, it was speculated that PA may have exacerbated cell death by somehow antagonizing excess α2β1 receptors. This hypothesis was further supported by data showing that constant expression of the α2β1 receptors in HepaRG cells was associated with constant PA-induced cell death. Additionally, the data from gene expression analysis confirmed the established link between activated caspase 8 and cleavage of BID [31]. This may explain the adaptive increase in BID gene expression, as most of it is cleaved to amplify mitochondrial-driven apoptosis seen in PA-treated HepG2 cultured on collagen I. As described elsewhere [20,41,42,43], our findings support the crosstalk involving amplified antagonized α2β1 receptors, increased apoptosis, reduced levels of P-FAK, and increased gene expression of TP53 and CDKN1B.

Since integrin-mediated death has not been described in NAFLD [44], the findings from this study imply that PA-induced lipotoxicity was incorrectly enhanced by culturing HepG2 on collagen I. Moreover, the data from this study could suggest that the EC50 and IC50 of some drugs tested in HepG2 in the presence of collagen I may be overestimated, especially if those drugs interact with integrin receptors. Therefore, researchers may need to compare the effects of drug agents in the absence or presence of collagen I in HepG2 or other cell lines. From a cancer research perspective, the findings from this study demonstrate that PA has the potential to antagonize integrin receptors, and its structure may be harnessed to design and develop drug agents against tumors with high levels of integrins, especially since integrin inhibitors are vital for cancer treatment [45,46].

Although the data from this study suggest that integrin-mediated death is likely responsible for the outcomes observed, future research is necessary to provide robust evidence by overexpressing or knocking down the ITGA2 or ITGB1 subunits in HepG2 to determine the effects of PA-induced cell death. In addition, future studies may also determine how PA interacts with α2β1 receptors using fluorescent or radiolabeled PA and receptor subunits.

## 5. Conclusions

In conclusion, the present study demonstrated for the first time that collagen I-cultured HepG2 exhibited increased cell death following exposure to PA through the mechanism of integrin-mediated death. The findings from this study may serve as a precaution to those using 2D models or 3D scaffold-based models of HepG2 in the presence of collagen I.

## Data Availability

Data are available from T.E.M. and O.K. upon request. E-mail: masekot@lfhk.cuni.cz (T.E.M.); kucerao@lfhk.cuni.cz (O.K.).

## Supplementary Materials

The following supporting information can be downloaded at: https://www.mdpi.com/article/10.3390/biom14091179/s1, Supplementary Figure S1: (A) Scheme of cell culture and application of treatments. (B) Relative gene expression of ITGA2 and ITGB1 following palmitate (PA) exposure in HepG2 cultured on collagen I for 48 hrs. (C) Cell viability of HepG2 pretreated (30 min) and cocultured with caspase inhibitors: [caspase 8, Z-IETD-FMK (Casp 8)], [caspase 9, Z-LEHD-FMK TFA (Casp 9)], and [caspase 3, Z-DEVD-FMK (Casp 3)]. 0.2% DMSO was used as a vehicle control. (D) Triacylglycerol (TAG) accumulation following PA exposure in HepG2 cultured in the absence or presence of collagen I for 48 hrs. Data are expressed as mean ± SD. Statistical analyses were carried out using two-way ANOVA followed by Tukey’s post-hoc test. ** *p* < 0.01, *** *p* < 0.001. ns, not significant. (A) *n* = 3, (B) *n* = 4, (C) *n* = 8, and (D) *n* = 4. O.D: optical density, BSA: bovine serum albumin. − Collagen I: absence, and + Collagen I: the presence of collagen I.
