# Supplementary material for: Collagen I Increases Palmitate-Induced Lipotoxicity in HepG2 Cells via Integrin-Mediated Death
Source: Biomolecules. 2024 Sep 20;14(9):1179. doi: 10.3390/biom14091179 (PMC11430893; doi:10.3390/biom14091179)
Supplement: Supplementary file 1 [file biomolecules-14-01179-s001.zip › Supplementary Materials and Methods.pdf]

Data S1: Supplementary Materials and Methods

Cell viability

Cell viability assay, WST-1 (4-[3-(4-iodophenyl)-2-(4-nitrophenyl)-2H-5-tetrazolio]-1,3-benzene disulfonate) purchased from Roche, Ltd was used to assess the effect of caspase inhibitors on PA-induced cell death (see the main article for a brief die. Following 24 hrs. of seeding, cells were treated for 30 min with 20 μM of inhibitors: [Z-DEVD-FMK, caspase 3, (Casp 3)], [Z-IETD-FMK, caspase 8, (Casp 8)], and [Z-LEHD-FMK TFA, caspase 9, (Casp 9)]. After that, cells were exposed to a combination of treatments (2.5% BSA and 1 mM PA) with the caspase inhibitors for a total culture period of 32 and 48 hrs. 10 mM stock solutions of inhibitors were prepared in dimethyl sulfoxide and stored at −20°C. All caspase inhibitors were purchased from MedChemExpress LLC.

Triacylglycerol (TAG) accumulation

The TAG content was quantified after 48 hrs. of culture using a TAG assay kit (Cayman Chemical Company) per the manufacturer's instructions. The attached cells were harvested from the cell culture plates using cell scrappers (Kisker Biotech GmbH & Co. KG), and detached cells were collected (940 × g, 4°C, 5 min) from the culture media. Subsequently, all collected cells were sonicated and stored at −80°C until spectrophotometric analyses (Tecan Infinite M200, Tecan Group, Ltd.).

Figure S1: (A) Scheme of cell culture and application of treatments. (B) Relative gene expression of ITGA2 and ITGB1 following palmitate (PA) exposure in HepG2 cultured on collagen I for 48 hrs. (C) Cell viability of HepG2 pretreated (30 min) and cocultured with caspase inhibitors: [caspase 8, Z-IETD-FMK (Casp 8)], [caspase 9, Z-LEHD-FMK TFA (Casp 9)], and [caspase 3, Z-DEVD-FMK (Casp 3)]. 0.2% DMSO was used as a vehicle control. (D) Triacylglycerol (TAG) accumulation following PA exposure in HepG2 cultured in the absence or presence of collagen I for 48 hrs. Data are expressed as mean ± SD. Statistical analyses were carried out using two-way ANOVA followed by Tukey's post-hoc test. ** $p < 0.01$, *** $p < 0.001$. ns, not significant. (A) $n = 3$, (B) $n = 4$, (C) $n = 8$, and (D) $n = 4$. O.D: optical density, BSA: bovine serum albumin. – Collagen I: absence, and + Collagen I: the presence of collagen I.
