# Supplementary figures and images for: Collagen I Increases Palmitate-Induced Lipotoxicity in HepG2 Cells via Integrin-Mediated Death
Source: Biomolecules. 2024 Sep 20;14(9):1179. doi: 10.3390/biom14091179 (PMC11430893; doi:10.3390/biom14091179)

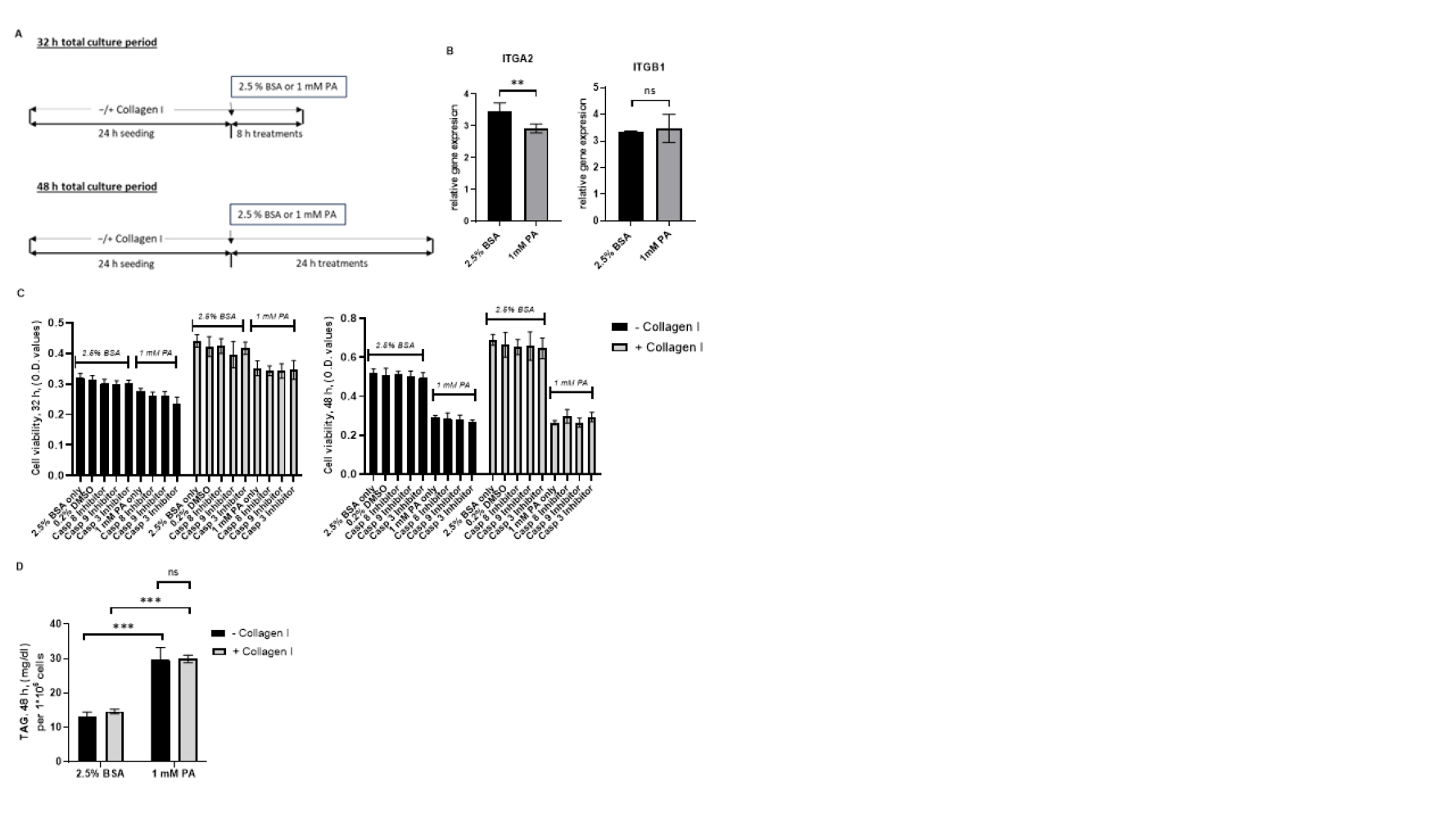

Supplement: Supplementary file 1 [file biomolecules-14-01179-s001.zip › Figure S1.tif]
